# Discovery of mosquitocides from fungal extracts through a high-throughput cytotoxicity-screening approach

**DOI:** 10.1186/s13071-021-05089-3

**Published:** 2021-12-04

**Authors:** Liang Jin, Guodong Niu, Limei Guan, Julian Ramelow, Zhigao Zhan, Xi Zhou, Jun Li

**Affiliations:** 1 Department of Biological Sciences, Florida International University, Miami, FL 33199 USA; 2 Institute of Microbiology, Jiangxi Academy of Sciences, Nanchang, Jiangxi China; 3 Biomolecular Sciences Institute, Florida International University, Miami, FL 33199 USA; 4 Herbert Wertheim College of Medicine, Florida International University, Miami, FL 33199 USA; 5 Wuhan Institute of Virology, Chinese Academy of Sciences, Wuhan, 430071 Hubei China

**Keywords:** Mosquito, Vector-borne diseases, Pesticides, *Penicillium toxicarium*, *Anopheles gambiae*

## Abstract

**Background:**

Mosquitoes transmit a variety of diseases. Due to widespread insecticide resistance, new effective pesticides are urgently needed. Entomopathogenic fungi are widely utilized to control pest insects in agriculture. We hypothesized that certain fungal metabolites may be effective insecticides against mosquitoes.

**Methods:**

A high-throughput cytotoxicity-based screening approach was developed to search for insecticidal compounds in our newly established global fungal extract library. We first determined cell survival rates after adding various fungal extracts. Candidate insecticides were further analyzed using traditional larval and adult survival bioassays.

**Results:**

Twelve ethyl acetate extracts from a total of 192 fungal extracts displayed > 85% inhibition of cabbage looper ovary cell proliferation. Ten of these 12 candidates were confirmed to be toxic to *Anopheles gambiae* Sua5B cell line, and six showed > 85% inhibition of *Anopheles* mosquito cell growth. Further bioassays determined a LC_50_, the lethal concentration that kills 50% of larval or adult mosquitoes, of 122 µg/mL and 1.7 µg/mosquito, respectively, after 24 h for extract 76F6 from *Penicillium toxicarium*.

**Conclusions:**

We established a high-throughput MTT-based cytotoxicity screening approach for the discovery of new mosquitocides from fungal extracts. We discovered a candidate extract from *P. toxicarium* that exhibited high toxicity to mosquito larvae and adults, and thus were able to demonstrate the value of our recently developed approach. The active fungal extracts discovered here are ideal candidates for further development as mosquitocides.

**Graphical abstract:**

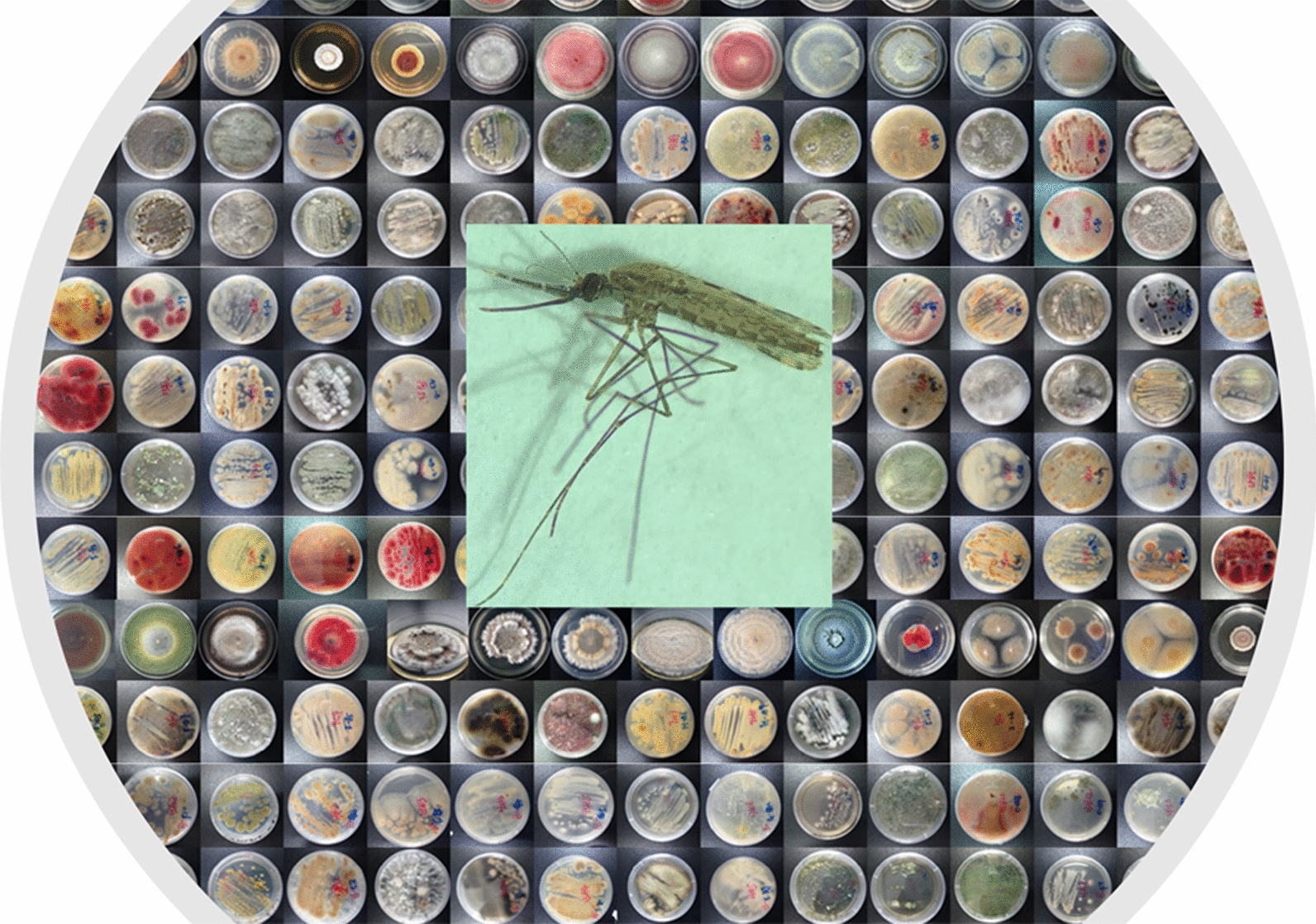

## Background

Mosquitoes transmit a variety of diseases such as malaria, dengue fever, and Zika virus. Malaria alone was responsible for approximately 409,000 deaths in 2019, according to a recent report from the World Health Organization. For decades, vector control and elimination strategies have been at the forefront of approaches used to decrease mosquito populations, interrupt the transmission cycles of vector-borne diseases, and reduce their occurrence [1–3]. Formerly, the highly controversial compound dichlorodiphenyltrichloroethane (DDT) was one of the prime pest control agents used in the fight against malaria [4]. Nowadays, DDT can only be used under very specific and critical conditions due to the harm it causes to the environment. Because of the limited number of mosquito molecules targeted by currently available insecticides, and the small number of available types of insecticide [5], insecticide resistance in mosquito populations has begun to accelerate worldwide, and is a major problem for malaria control [6]. In addition, only a few novel insecticides have been introduced into the market during the past 30 years [7, 8]. Therefore, researchers and public health authorities are eagerly awaiting the discovery of novel insecticides to enable the control of malaria vector populations with a high rate of success.

Natural resources are commonly exploited for novel drug research and development, and some insecticides have been discovered from plants and fungi [9, 10]. Pyrethrin, for example, which was identified from the plant *Chrysanthemum cinerariifolium* and subsequently used for the development of pyrethrin analogs (synthetic pyrethroids), has proven to be highly useful for the successful treatment of many insect pests [11]. Fungi offer many advantages over plants as sources of metabolites because (i) an enormous number of fungal species have been identified, and many more await discovery; (ii) they produce diverse secondary metabolites; (iii) their metabolites can be generated using large-scale fermentation approaches, which makes them very attractive for further development [12–14].

Several studies have reported that species of some fungal genera, e.g. *Lagenidium*, *Coelomomyces*, *Conidiobolus*, *Entomophthora*, *Culicinomyces*, *Erynia*, *Beauveria*, and *Metarhizium*, display a potent ability to kill many species of mosquitoes, including those of the genera *Anopheles*, *Culex*, and *Aedes* [15, 16]. Entomopathogenic fungi have also been widely utilized for the treatment of insect pests, especially in agriculture. Their life cycles are associated with the synthesis and secretion of different active metabolites, such as destruxins, efrapeptins, oosporein, beauvericin, and beauveriolides. The effects of these types of compounds on insects have been summarized by Strasser et al. [17]. Mycelial extracts of various fungi showed high cytotoxicity and toxicity to larval and adult stages of mosquitoes [18, 19]. Based on the available literature, we hypothesize that new, effective insecticides can be produced from fungal metabolites.

To date, the discovery of natural fungal metabolites that are insecticidal has been challenging, as very time-consuming bioassays are the most effective means of testing fungal samples on live insects. An efficient high-throughput screening approach is not yet available for this [20]. Additionally, the lack of a publicly accessible library of diverse fungal metabolite extracts contributes to this lack of discovery of new insecticides.

Our lab has recently established a large and diverse global fungal extract library (GFEL), which contains more than 10,000 fungal isolates and many more structurally diverse metabolites [21]. This library includes metabolites from some fungal species, e.g. *Penicillium* spp., *Aspergillus* spp., *Fusarium* spp., *Podospora* spp., *Mucor* spp., *Cladosporium* spp., and *Stoloniferum* spp. which have been reported to possess larvicidal and adulticidal activities [15, 16]. The GFEL provides researchers with an important resource for the discovery of novel insecticides and ultimately for the control of mosquito populations.

To overcome the time-consuming nature of bioassays, we developed a new high-throughput screening approach that is based on cytotoxic activity assays against major insect cell lines. This new approach allows the determination of candidate fungal extracts that have exhibited toxic properties in preliminary tests. Following the discovery of these candidate extracts, we then validated their insecticidal effects by using traditional bioassays against larval and adult stages of a mosquito. Our results demonstrate that this newly developed assay can be utilized for the initial screening of fungal extracts for metabolites that have potential as novel insecticides.

## Methods

### Rearing *Anopheles gambiae*

*Anopheles gambiae* G3 strain was obtained from BEI Resources (BeiResources.org). The strain was originally collected on McCarthy Island, Gambia, West Africa. It is a wild-type mosquito strain that has been used in research for more than 30 years. *Anopheles gambiae* G3 strain mosquitoes were reared in a closed Darwin growth chamber at constant 27 °C and 80% humidity under a 12-h light/12-h dark cycle and standard laboratory conditions. Mosquito larvae were fed with 0.05 mg ground fish food for nishikigoi (Hikari, Japan) per larva per day. The adult mosquitoes were kept on 10% sucrose and were occasionally blood-fed with human blood and serum obtained from a blood bank (Oklahoma Blood Institute, Oklahoma City, OK) for egg production.

### Cell culturing

We used the BTI-Tn-5B1-4 cell line [High Five Cells (Hi-5)], which is derived from ovarian cells of the cabbage looper (*Trichoplusia ni*) and is the standard insect cell line commonly used in many laboratories. We also chose to use the *An. gambiae* Sua5B cell line as these cells are immunocompetent and hemocyte-like. The Hi-5 and Sua5B cells were cultured in Express Five SFM medium (Invitrogen) and Grace’s Supplemented Insect Medium (Gibco, Waltham, MA), respectively, at 27 °C, as described previously [22]. The Sua5B cell medium also contained 10% heat-inactivated fetal bovine serum (Gibco); the cell lines were generally passaged twice per week upon reaching 90% confluency.

### Fungal culture and metabolite extraction process

A global fungal library was recently generated and reported in the literature [21]. In summary, 2395 soil samples and 2324 plant samples were collected from 36 regions of Africa, Asia, and North America. About 10,000 fungal strains were isolated from these samples. The fungi were cultured on a large scale, as described previously in detail [23]. In short, the fungi were cultured with 500 g of Cheerios breakfast cereal (General Mills, Minneapolis, MN). The cereal was sterilized, dried, and mixed with 1 L of sterile 0.3% sucrose solution containing 0.005% chloramphenicol. The fungal cultures were incubated at 27 °C for 4 weeks in a mushroom bag, and fungal metabolites in the solid culture were extracted with 2 L of ethyl acetate. Ethyl acetate enables the extraction of only small molecules, and large molecules such as DNA and proteins are eliminated.

### MTT assays for cell survival measurement

MTT is a compound that acts as a hydrogen ion acceptor in the respiratory chain in the mitochondria of living cells. After entering a living cell, MTT is reduced into formazans, which are water-insoluble blue-purple crystalline structures that are deposited in the cell. It is important to note that this reaction does not occur in dead cells, and that dimethyl sulfoxide (DMSO) can dissolve formazans in cells. The light absorption at 570-nm wavelength is used to quantify formazan deposits. Thus, we deduced that the absorbance level at 570 nm corresponded to the number of living cells [24, 25].

The initial screening of the extracts was carried out with Hi-5 cells. About 2 × 10^4^ Hi-5 cells were seeded in each well of a TC-treated 96-well plate (Corning, NY). After cell attachment, the initial seeding medium was removed and replaced with 99 µL of fresh Express Five SFM medium (Invitrogen). About 1 µL of fungal extract at 100 µg/mL final concentration dissolved in DMSO was added to each well. A 1-µL volume of DMSO was used as the negative control, to show that it did not kill the cells. We chose a lethal dosage of blasticidin, a peptidyl nucleoside antibiotic isolated from *Streptomyces griseochromogenes* that inhibits protein synthesis, at a final concentration of 50 µg/mL as the positive control. At this concentration, blasticidin is known to kill 100% of Hi-5 and Sua5B cells. The plates were then incubated at 27 °C for 24 h. Next, 10 µL of MTT dissolved in PBS at a concentration of 5 mg/mL was added to each well and incubated for 4 h at 37 °C with a 5% CO_2_ supply. The medium was then removed from each well and 100 µL acidic isopropanol (0.04 N HCl in isopropanol) solution was added and mixed thoroughly. The plate was incubated again for 10 min at 37 °C to dissolve formazan crystals. Absorbance measurement at 570 nm was carried out with an Epoch Microplate Spectrophotometer (BioTek, Winooski, VT). The data were analyzed with Prism 9.2 software using the ANOVA test (GraphPad, San Diego, CA). The following equations were used:

i. To calculate the inhibition activity of a single fungal extract on cell proliferation,
  $${\text{Extract inhibition activity}} = \frac{{{\text{A}}_{570} {\text{ DMSO}} - {\text{A}}_{570} {\text{ Experimental}}}}{{{\text{A}}_{570} {\text{ DMSO}} - {\text{A}}_{570} {\text{ Blasticidin}}}} \times 100$$;
ii. To calculate the cell survival rate,
  $${\text{Cell survival rate}} = \frac{{{\text{A}}_{570} {\text{ Experimental}} - {\text{A}}_{570} {\text{ Blasticidin}}}}{{{\text{A}}_{570} {\text{ DMSO}} - {\text{A}}_{570} {\text{ Blasticidin}}}} \times 100$$.

As a subsequent validation step and to further confirm the cytotoxicity of candidates as potential mosquitocides, Sua5B mosquito cells were seeded at 2 × 10^4^ per well and used in another MTT assay at 100 μg/mL final concentration. The overall procedure and analysis for the Sua5B assay were the same as those described above for the Hi-5 cells.

### Larvicidal bioassays

Larval mortality bioassays were carried out according to standard protocols provided by the World Health Organization [26] with only slight modifications [27]. First, approximately 10 mL of distilled water was added to a 50-mL beaker. Then, each fungal extract dissolved in DMSO was added to 60-mm × 15-mm Petri dishes at final concentrations of 0, 50, 100, 200, 300, and 400 µg/mL. About 20 fourth-instar larvae (L4) were transferred to the Petri dishes and incubated for 24 h in a closed Darwin growth chamber at constant 27 °C and 80% humidity under a 12-h light/12-h dark cycle and standard laboratory conditions. Ground fish food was not supplied during this stage of the experimental process. Finally, the live and dead mosquito larvae were counted for each Petri dish and the following equation was used to determine extract toxicity:

$${\text{Toxicity}} \left( \% \right) = \frac{{{\text{Mortality rate of experimental}} - {\text{control mortality}}}}{{100 - {\text{control mortality}}}} \times 100$$

Three replicates per dose, including the negative control (1% DMSO without any fungal extract), were used in the same experimental setting, and three independent repeats were carried out for the dose–response assays. The lethal concentration of the extracts that killed 50% of the larvae (LC_50_) was calculated using Prism 9.2 (GraphPad Software, CA).

### Synergetic effects of piperonyl butoxide on fungal candidate toxicity

We tested for a synergetic effect between the fungal extract candidates and piperonyl butoxide (PBO), a known cytochrome P450 inhibitor of the detoxification pathway in insect cells [36]. Firstly, the sub-lethal concentration of PBO against the larvae was examined by testing final concentrations of 1, 3, 4, 5, 10, 50, and 100 µg/mL. *Anopheles gambiae* L4 larvae were sorted into glass Petri dishes filled with distilled water to which PBO was added at the above concentrations. Larval mortality was determined 24 h post-treatment. Next, 76F6 alone or 76F6 (both at 125 μg/mL final concentration) supplemented with PBO at the maximum sublethal dose was added to the distilled water. Water containing only PBO was used as the negative control. Next, 20 L4 larvae from each group were transferred into the Petri dishes and maintained in a closed Darwin growth chamber at constant 27 °C and 80% humidity under a 12-h light/12-h dark cycle and standard laboratory conditions. Mortality was recorded after 24 h and the experiments were repeated three times.

### Pesticide bioassays for adult mosquitoes

Based on a recent approach to test pesticides via bioassays for adult mosquitoes [28], we determined the toxicity of candidate fungal extracts against adult female *An. gambiae*. *Anopheles gambiae* were maintained ad libitum on a sterile 10% sucrose solution. Naïve 3- to 5-day-old female mosquitoes were cold-anesthetized on ice and sorted into groups of 24 in a glass Petri dish to give experimental and control groups. About 0.5 µL of the fungal extract in acetone at a concentration of 5 µg/µL was deposited on the notum of a mosquito. Approximately 0.5 µL acetone was used as a control. There were about 12 female *An. gambiae* in each group. After successful application of the fungal extracts, the mosquitoes were transferred to a 5-ounce Solo waxed paper water cup (Dart Container, MI) and maintained on the sterile 10% sucrose diet.

To assess a possible dose-dependent response of the fungal extracts, new mosquitoes were cold-anesthetized on ice and sorted into groups of 12 females in a glass Petri dish, as described earlier. We dissolved the extracts in acetone to obtain final concentrations of 0.25, 0.5, 1, 2.5, 5, and 10 µg/mosquito, and applied the extracts to the nota, as described earlier. Post-treatment, the mosquitoes were transferred to a 5-ounce waxed paper cup and maintained on the sterile 10% sucrose diet. Mortality was recorded after 24 h. The experiments were conducted with triplicates per sample and performed as three independent repeats.

### Identification of fungal species

The conserved sequences of the internal transcribed spacer (ITS) region of 5.8S and 28S ribosomal DNA were used to identify individual fungal species [21]. A small amount of mycelium (0.1–1 mg) was taken from fungus 76F6, rinsed in 400 μL sterilized water, and then collected by centrifugation at 15,000 *g* for 2 min. The mycelium was re-suspended in 100 μL sterilized water, 1 μL of which was used for PCR. The DNA fragments were amplified using ITS1 (TCCGTAGGTGAACCTGCGG) and ITS4 (TCCTCCGCTTATTGATATGC) primers [21]. The reaction was carried out under the following conditions: 94 °C for 2 min to denature DNA; 35 cycles of 94 °C for 30 s, 55 °C for 30 s, and 72 °C for 1 min; and 72 °C for 5 min to complete the reaction. The amplified PCR product was gel-extracted, purified, and sequenced (Eurofins Genomics, Louisville, KY). The raw sequence data were analyzed, and low-quality sequence ends removed. Subsequently, the edited sequences were compared with the National Center for Biotechnology Information database using BLAST to identify individual fungal species. Based on the ITS sequences, a phylogenetic tree was constructed via the neighbor-joining method using MEGA 6.0 software, as described previously [29].

### Statistical analysis

*P*-values, LC_50_, LC_90_, confidence intervals, and slopes were calculated using previously reported approaches [30–32] implemented in Prism 9.2 software (GraphPad Software).

## Results

### High-throughput MTT-based cytotoxicity assay screening for fungal extracts that inhibited cell survival

Our MTT-based cytotoxicity assay for high screening purposes was used to find insecticide candidates by quantifying living cells. Thus, we initially tested our GFEL for insecticide candidates on Hi-5 cells. Experimental optimization via MTT assays determined the toxic effects of the extracts to occur at a concentration of 100 μg/mL. Hence, 100 μg/mL was used as a standard concentration for our screening assays.

**Fig. 1:**
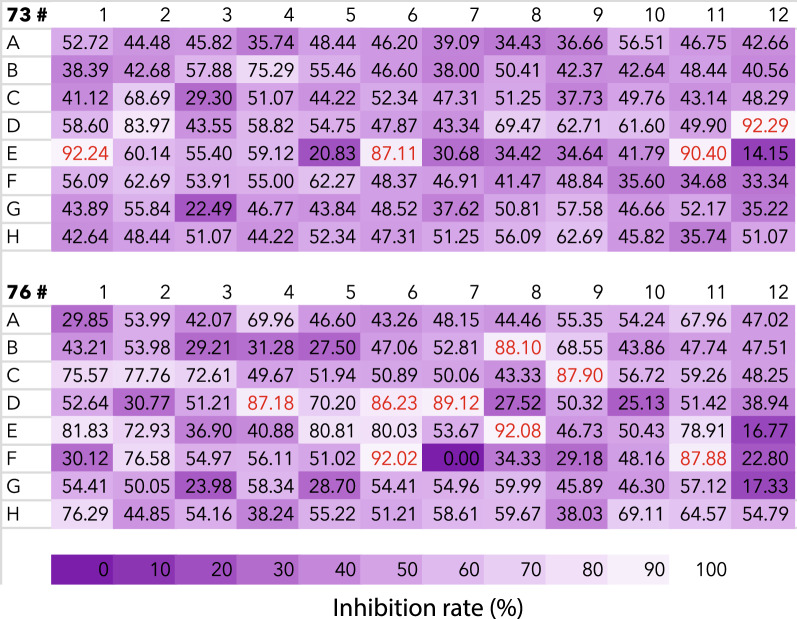
Toxicity of the ethyl acetate extracts of the different fungi (100 μg/mL) against High Five Cells (Hi-5). The numbers indicate percentage inhibition of cell proliferation. Lighter purple colors indicate higher inhibition, darker purple colors indicate lower inhibition, and red font colors highlight >85% inhibition rate

**Fig. 2:**
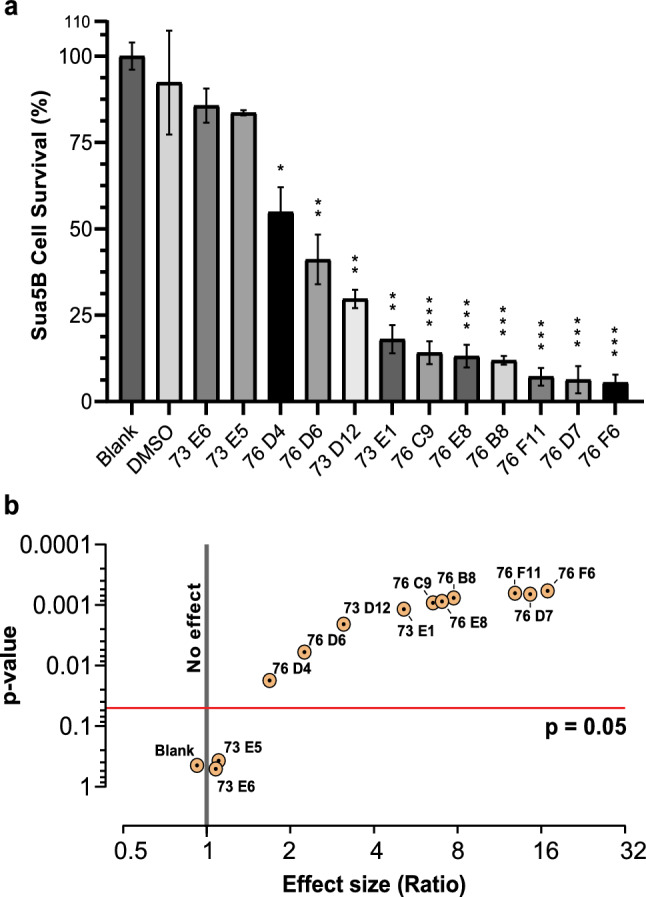
**Sua5B cell survival analysis of the twelve candidate fungal extracts.** *Anopheles gambiae* Sua5B cell line (*Sua5B*) cell survival analysis for the 12 candidate fungal extracts. **a** Ten extracts significantly decreased the survival of Sua5B cells when compared to the negative control, dimethyl sulfoxide (*DMSO*), by *t*-test. There were three replicates for each treatment. **b** Effect size (fold change) of the individual extracts against Sua5B cells (log^10^ *y*-axis) with respective *P*-values. *P* = 0.05 served as the cutoff for statistical significance; *** P < 0.001, ** P < 0.01, * *P* < 0.05

Our results demonstrated that, of 192 fungal extracts, 12 candidates had a > 85% inhibition rate for Hi-5 cells (Fig. 1). These fungal extracts were 73D12, 73E1, 73E6, 73E11, 76B8, 76C9, 76D4, 76D6, 76D7, 76E8, 76F6, and 76F11. These extracts were chosen for additional screening using mosquito Sua5B cells. Ten of these extracts significantly decreased the survival of Sua5B cells when compared to the negative control, DMSO (Fig. 2a; *P* < 0.05). Six of the extract candidates (76B8, 76C9, 76D7, 76E8, 76F6, and 76F11) exhibited very high toxicity, as only < 20% of Sua5B cells survived. The *P*-value was calculated for the effect of each fungal extract versus the DMSO control. Effect size vs *P*-value showed that these six candidate extracts indeed had a large effect on cell survival rate, with a ratio of > 6 (Fig. 2b; *P* < 0.001). Due to their high cytotoxicity and deleterious effect on cell survival, these six candidate insecticides were analyzed further.

**Fig. 3:**
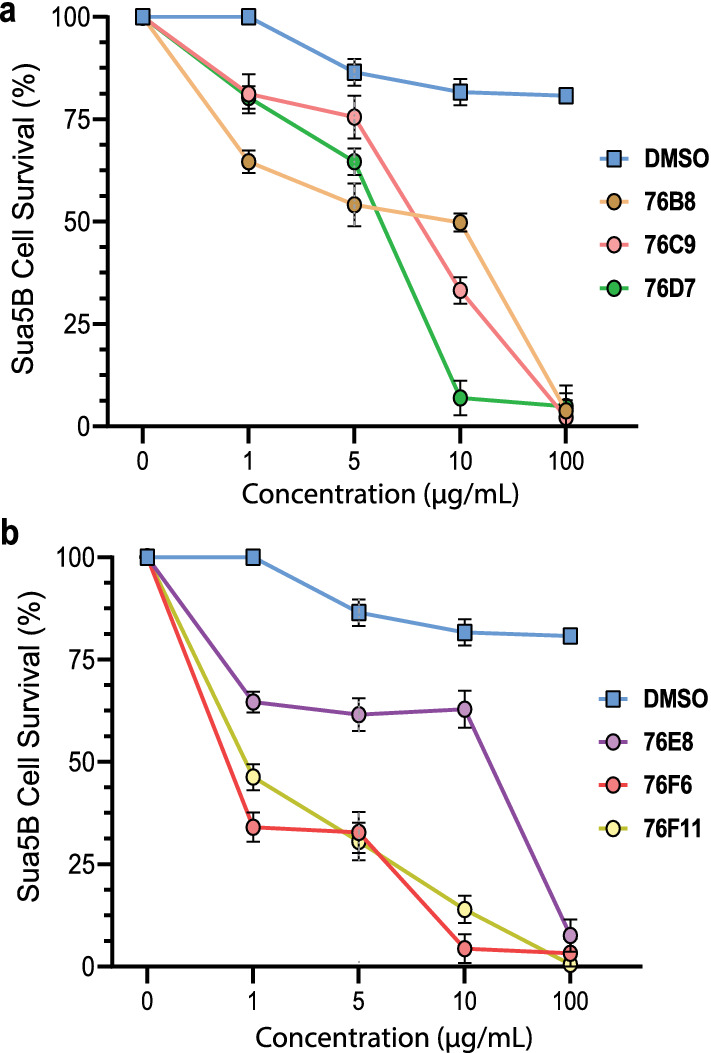
The six selected candidate fungal extracts exhibited dose-dependent effects against Sua5B cell survival. For abbreviations, see Fig. 2

We also measured the effects of different fungal extract concentrations on Sua5B with our MTT-based cytotoxicity assay. The toxic effects were highly dose-dependent, as elevated concentrations displayed increased toxicity and lower survival rates of cells when compared to the control, DMSO (Fig. 3a). Extracts 76F6 and 76F11 showed the most potent cytotoxicity, even at a low concentration of 1 μg/mL (Fig. 3b). At a final concentration of 100 μg/mL, all the extracts showed nearly complete inhibition of Sua5B cell growth. The half-maximal inhibitory concentration (IC_50_) of 76B8, 76C9, 76D7, 76E8, 76F6 and 76F11 was 12.3 μg/mL, 8.2 μg/mL, 5.7 μg/mL, 42.2 μg/mL, 0.47 μg/mL, and 1.0 μg/mL, respectively. We concluded that, among the candidate fungal extracts, 76F6, which had an IC_50_ of 0.47 μg/mL, was the most effective in inhibiting the proliferation of Sua5B cells.

### Candidate fungal extracts exhibited lethal effects on mosquito larvae

**Fig. 4:**
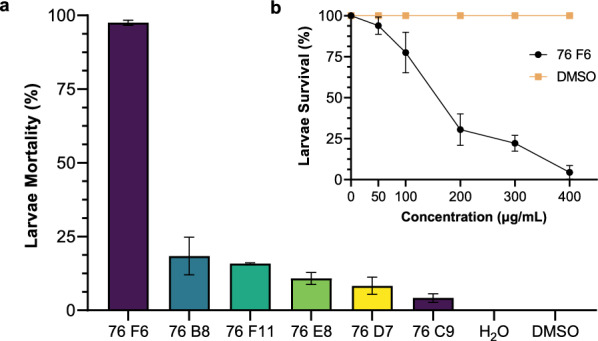
Larvicidal activity of candidate fungal extracts against fourth-instar *Anopheles gambiae* 24 h post-exposure. **a** The larvicidal activity of different fungal extracts (400 μg/mL) based on three replicates. **b** The larvicidal activity of fungal extract 76F6 was measured in triplicate and was dose-dependent

We further analyzed the six crude fungal extract candidates and investigated their larvicidal activities to determine their potential as possible insecticides. Fourth-instar larvae of *An. gambiae* were treated in distilled water containing one of the six fungal extracts at 400 μg/mL final concentration. These fungal extracts displayed various larvicidal activities (Fig. 4a). The toxicity of the 76F6 fungal extract at 400 μg/mL to mosquito larvae was close to 100%, while the toxicity of the other five fungal extracts against mosquito larvae was only < 40%. The experiments were repeated in triplicate and similar trends obtained. Next, we carried out a serial dilution of 76F6 from 50 μg/mL to 400 μg/mL final concentration. We determined that the effect of 76F6 was dose-dependent, and its LC_50_ and LC_90_ were 122 μg/mL and 295.8 μg/mL, respectively (Table 1; Fig. 4b). Thus, we can conclude that extract 76F6 is a prime fungal candidate for the control of *An. gambiae* larvae.

**Table 1:** Lethal concentration of fungal extract 76F6 that killed 50% of larval or adult stages of *Anopheles gambiae* (LC_50_) and LC_90_ values

Life cycle stage	LC_50_	95% CI		Slope	LC_90_	*R*^2^
		Upper	Lower			
Larvae	122^a^	1889^a^	7.3^a^	2.9 ± 0.36^a^	295.8^a^	0.98
Adult	1.7^b^	39.4^b^	0.07^b^	4.5 ± 0.92^b^	2.8^b^	0.97

For larvicide assays, each triplicate comprised 20 fourth-instar larvae and the experiments were repeated three times. For adult assays, each experimental or control group comprised 24 female 3- to 5-day-old mosquitoes. The experiments were conducted in triplicate and performed as three independent repeats

*R*^2^ Fitness,* CI* confidence interval

^a^μg/mL

^b^μg/mosquito

### Piperonyl butoxide shows synergetic toxic effects with 76F6 against *An. gambiae* larvae

**Fig. 5:**
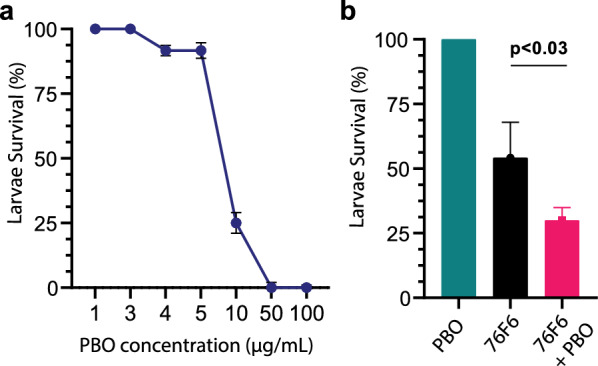
Synergetic effects of extract 76F6 and the cytochrome P450 inhibitor piperonyl butoxide (*PBO*) on mosquito larvae. **a** Larvae survival rates at various concentrations of PBO (*n* = 3). **b** In the presence of 3 μg/mL PBO, extract 76F6 (125 μg/mL) killed significantly more larvae than when applied alone (*P* < 0.03; *n* = 3)

We also evaluated the synergetic effect between extract 76F6 and PBO on the mortality of *An. gambiae* larvae. Cytochrome P450 enzymes detoxicate small molecular toxins and PBO is an inhibitor of these enzymes. Firstly, we performed a serial dilution of PBO in distilled water to determine the optimal sublethal dose for *An. gambiae* larvae where no larvae are killed. The maximum sublethal dosage of 76F6 alone was about 3 μg/mL, and PBO alone exhibited toxic effects at concentrations higher than 3 μg/mL (Fig. 5a). Next, the toxic effects of PBO and 76F6 in combination on *An. gambiae* larvae were examined. After a 24-h incubation, the survival rate of the 76F6-treated mosquito larvae in the presence of PBO was significantly decreased, from 54.2 to 29.9% (Fig. 5b; *P* < 0.03), thus the potency of 76F6 was higher in the presence of PBO. This indicates that synergetic compounds, such as PBO, can be used in combination with 76F6 to improve its toxicity against larvae. These results support our hypothesis that cytochrome P450s in *An. gambiae* participate in the detoxification of active small molecules in extract 76F6.

### Candidate fungal extracts also exhibited lethal effects on adult mosquitoes

**Fig. 6:**
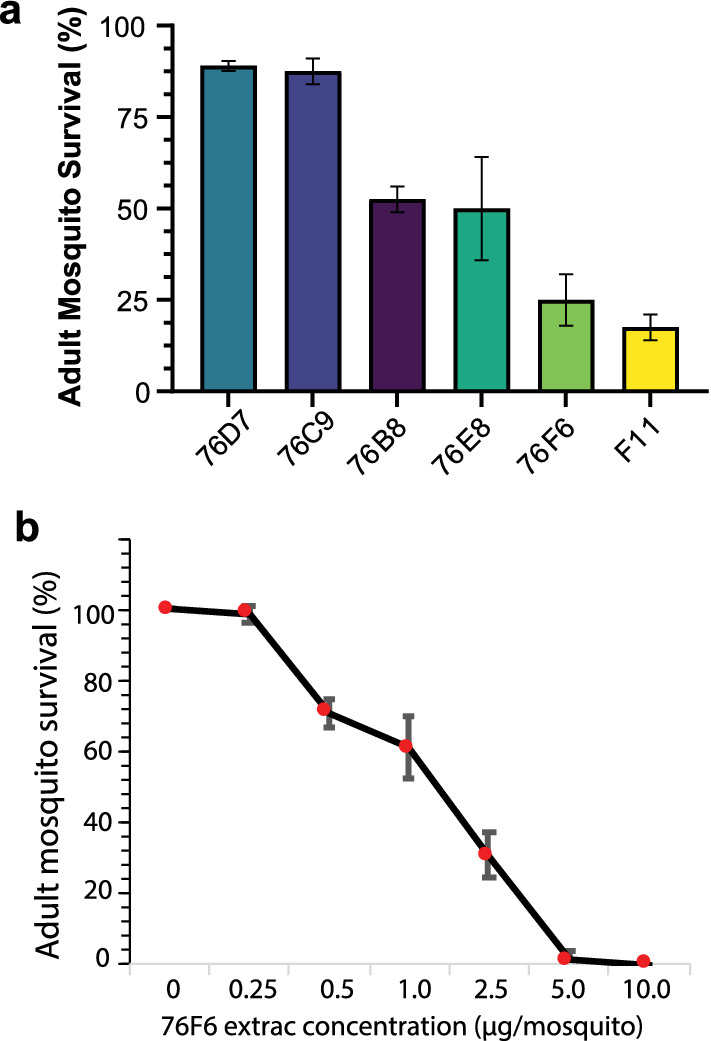
Survival rates of *Anopheles gambiae* adults after exposure to candidate fungal extracts. **a** Survival rates of 3- to 5-day-old adult *An. gambiae* treated with 2.5 μg/mosquito of the six tested extracts. **b** Dose-mortality curve of adult *An. gambiae* treated with extract 76F6. The experiments were conducted in triplicate and performed as three independent repeats. Data are means ± SEs

Following our discovery that fungal extract 76F6 could effectively kill mosquito larvae, we examined the effects of our six candidate fungal extracts on adult *An. gambiae*. Four of the six candidates caused below 50% survival after the application of 2.5 µg fungal extract/mosquito to the notum (Fig. 6a). Consistent with our earlier results demonstrating the high toxicity of fungal extract 76F6 to larvae, this extract greatly diminished adult mosquito survival to ~ 25%. Consequently, the toxicity of 76F6 at different concentrations was measured against adult *An. gambiae*. The results showed that the toxicity of 76F6 increased as its concentration increased (Fig. 6b). The LC_50_ of 76F6 on adult *An. gambiae* was 1.7 µg/mosquito, while the LC_90_ was 2.8 μg/mosquito (Table 1).

### Examination of fungal extract 76F6 reveals a relationship with *Penicillium toxicarium*

**Fig. 7:**
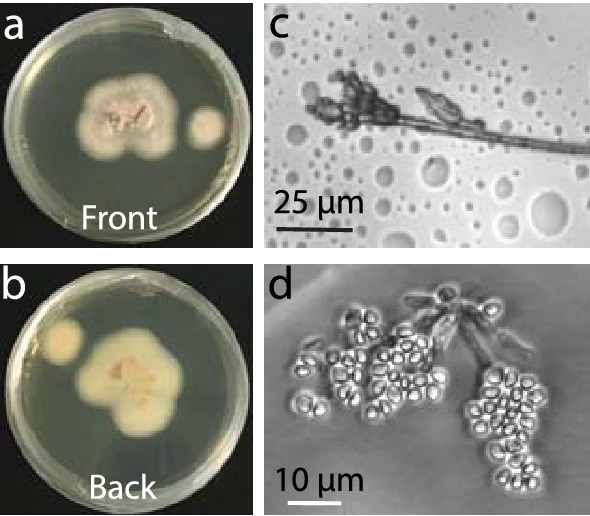
Morphology of candidate fungus 76F6. **a** Front view of a fungal colony. **b** Back view of a fungal colony. **c**, **d** Fungal conidia

We examined the morphology of several 76F6 fungal colonies on potato dextrose agar plates. After 7 and 30 days of incubation at 25 °C, the colonies attained a size of 50 mm and 65 mm in diameter, respectively. The colonies were slightly pink in pigmentation (Fig. 7a). Viewed from the reverse side of the plate, the colonies appeared to be light yellow in color (Fig. 7b). Conidia were present as long dry chains, and the conidiophores growing from the aerial mycelium were about 5 μM long (Fig. 7c). The mature conidia were round and approximately 2–4 μM in diameter (Fig. 7d). The morphology of this fungus was extremely similar to that of *Penicillium *spp*.* [33].

**Fig. 8:**
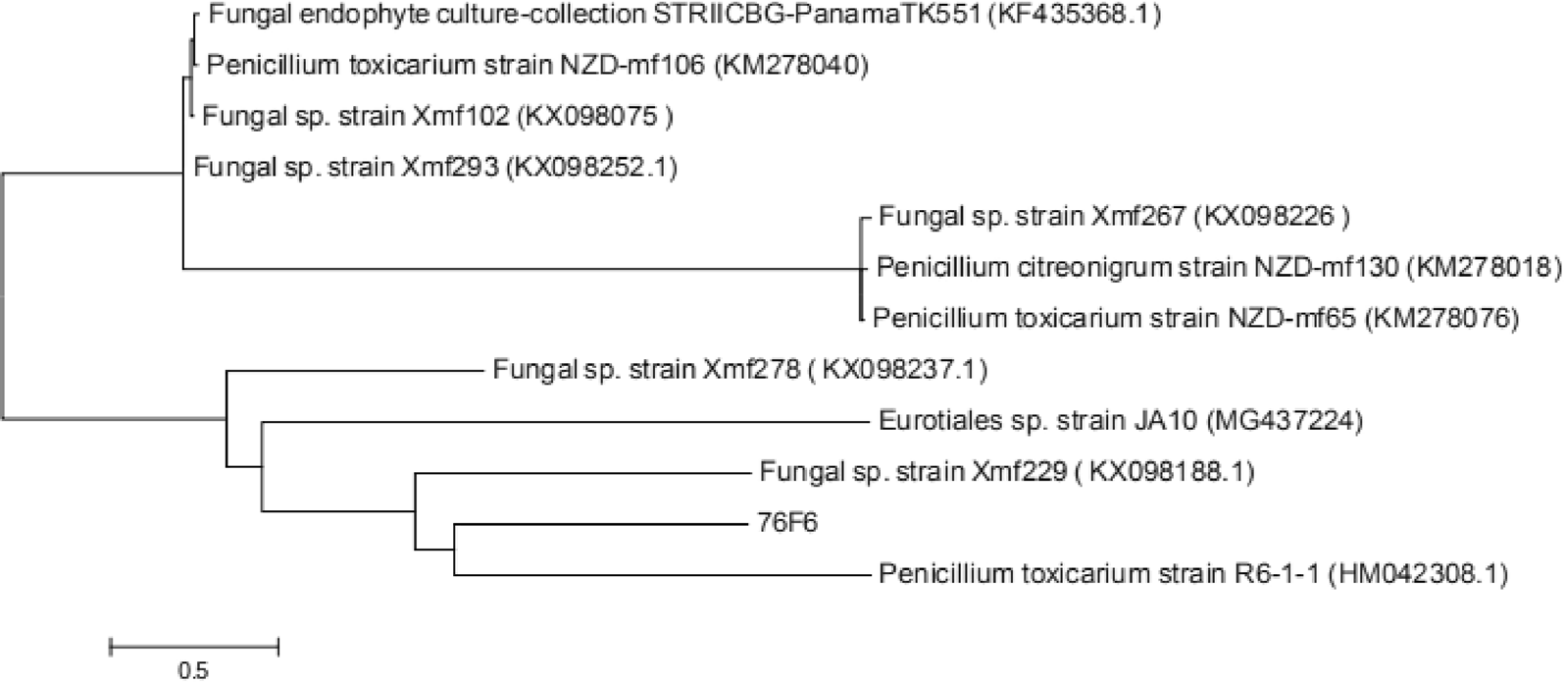
Phylogenetic analysis of the 18S ribosomal DNA sequences of the fungal strain from which extract 76F6 was obtained

Next, the ITS region of the 76F6 fungus was amplified and sequenced to identify the fungal strain and further verify its morphological similarity. The obtained ITS sequence has been submitted to GenBank (accession number MT072229). The BLAST tool indicated that the strain from which extract 76F6 was obtained is evolutionarily related to *Penicillium toxicarium*, with > 95% coverage and > 99% sequence identity (Fig. 8).

## Discussion

The increasing resistance of mosquitoes to insecticides advocates for the discovery of novel mosquitocides. However, it is currently extremely difficult to screen a large number of samples within a short time using traditional bioassays. We established a high-throughput cytotoxicity assay against Hi-5 and Sua5B insect cells that was based on the original MTT assay to screen our fungal extract library for insecticidal potential [24]. The results showed that our MTT-based high-throughput cytotoxicity assay is a useful alternative to standard bioassays for the initial screening and identification of cytotoxic extracts used for downstream analysis. Hi-5 cells are commonly available in laboratories and generally used for protein expression, but also serve as an important model for mechanistic studies on a variety of insecticidal substances [34]. Hence, we used Hi-5 cells during the initial screening round. The subsequent cytotoxicity analysis of the candidate fungal extracts against Sua5B, a mosquito cell line that originates from *An. gambiae* hemolymph, was important to assess their effectivities as mosquitocides. These results also narrowed the list of candidates for the subsequent bioassays, which were very time-consuming. Finally, we identified 76F6 as a potential candidate for larval control from our initial MTT-based high-throughput cytotoxicity assay.

PBO is a known inhibitor of cytochrome P450 monooxygenases (P450s) [35]. P450s are well known for their abilities to detoxify many different insecticides against mosquitoes, including pyrethroids, DDT, and some organophosphate insecticides [36]. We additionally showed that, upon the addition of PBO, the toxicity of 76F6 increased significantly, indicating that the active compound in extract 76F6 is possibly a small molecule that may well pass through a cell’s detoxifying pathway. The exact pathway of this compound and how it exhibits synergistic toxic effects with the addition of PBO remain unknown and thus need to be investigated in future studies.

In this study, we ultimately discovered that four of the fungal extract candidates were able to kill more than half of the adult mosquitoes at a dose of 2.5 µg/mosquito. It is noteworthy that fungal extract 76F6 exhibited a potent toxic effect on both mosquito larvae and adults, whereas extracts 76B8, 76E8, and 76F11 only had a very small effect on L4 larvae. However, these latter extracts showed medium-to-high toxic activity in adult mosquitoes. The reasons for this divergence may be the fluctuating stability of active compounds in water, the differential expression of targets in larval stages, the entry of active components into larvae, or the activity of detoxification mechanisms in larvae and adult mosquitoes. Future studies are required to determine the exact active chemical structures of these compounds and if they can potentially exhibit higher toxicity to L4 larvae and adult mosquitoes at higher concentrations.

Fungal extract 76F6, from a strain of *P. toxicarium*, was shown, to our knowledge for the first time, to kill both larval and adult *An. gambiae*. The LC_50_ of this extract for the L4 larvae was about 122 μg/mL, which is very similar to that of an ethyl acetate extract of a strain of *Penicillium daleae* on L4 larvae of *Culex quinquefasciatus* [19]. The phylogenetic analysis showed that these two strains of *Penicillium* only shared 26.8% identical ITS sequences, suggesting that their active compounds may be different. For further identification of its active compounds, the chemical structure of fungal extract 76F6 needs to be elucidated.

The MTT-based high-throughput cytotoxicity-screening assay for pesticides that we present here requires less labor than the other available high-throughput approaches for larvae [37]. It also proved to be extremely useful for the discovery of new insecticide candidates.

## Conclusions

We successfully established a high-throughput MTT-based cytotoxicity screening approach to search for and discover new pesticides based on their cytotoxic activity. We successfully demonstrated the discovery of several mosquitocides via this method using our fungal extract library. One of the candidate insecticides, 76F6, an extract of *P. toxicarium*, was found to be a suitable candidate for mosquito control.

## Data Availability

All data generated or analyzed during this study are included in this published article. The ITS sequence is available from GenBank (accession number MT072229).
